# Cross-modal deactivations during modality-specific selective attention

**DOI:** 10.1186/1471-2377-8-35

**Published:** 2008-09-25

**Authors:** Jennifer L Mozolic, David Joyner, Christina E Hugenschmidt, Ann M Peiffer, Robert A Kraft, Joseph A Maldjian, Paul J Laurienti

**Affiliations:** 1 Wake Forest University School of Medicine, Medical Center Boulevard, Winston-Salem, NC 27157, USA

## Abstract

**Background:**

Processing stimuli in one sensory modality is known to result in suppression of other sensory-specific cortices. Additionally, behavioral experiments suggest that the primary consequence of paying attention to a specific sensory modality is poorer task performance in the unattended sensory modality. This study was designed to determine how focusing attention on the auditory or visual modality impacts neural activity in cortical regions responsible for processing stimuli in the unattended modality.

**Methods:**

Functional MRI data were collected in 15 participants who completed a cued detection paradigm. This task allowed us to assess the effects of modality-specific attention both during the presence and the absence of targets in the attended modality.

**Results:**

The results of this experiment demonstrate that attention to a single sensory modality can result in decreased activity in cortical regions that process information from an unattended sensory modality (cross-modal deactivations). The effects of attention are likely additive with stimulus-driven effects with the largest deactivations being observed during modality-specific selective attention, in the presence of a stimulus in that modality.

**Conclusion:**

Modality-specific selective attention results in behavioral decrements in unattended sensory modalities. The imaging results presented here provide a neural signature (cross-modal deactivation) for modality-specific selective attention.

## Introduction

Our perception of the environment is shaped by the combination of information from multiple sensory modalities. While there are distinct regions of the cortex devoted to processing information gathered by each of the sensory systems, areas once thought to be modality-specific are now known to be influenced by input from other sensory modalities. For example, activity in both the auditory and visual cortices can be amplified by contextually or spatially congruent multisensory stimuli [1-3]. Additionally, unisensory auditory, visual, and somatosensory stimuli that are known to activate the corresponding sensory cortex can also significantly decrease activity below baseline levels in sensory cortices that process information from modalities in which no stimulus is presented [4,5]. We use the term "cross-modal deactivations" to refer to these activity decreases that occur across sensory domains.

In addition to incoming sensory information, top-down modulation from frontal and parietal cortices can also influence activity in the sensory cortices [6-10]. Focusing attention on a particular spatial location or stimulus feature has been shown to enhance activity in regions of visual cortex responsible for processing that information, while suppressing activity in surrounding cortical areas [8,11,12]. Such modulations in cortical activity can even be observed in the absence of any stimuli, indicating that they can be produced solely by attentional effects independent of an actual stimulus [13-15].

These mechanisms of visual spatial attention have been studied extensively, however, less is known about the impact that attention to a single sensory modality has on sensory processing. Behavioral experiments demonstrate that the primary effect of modality-specific attention is decreased processing of stimuli in an unattended modality, while task performance in the attended modality experiences little to no improvement [16,17].

Imaging data consistently demonstrate that focusing attention on stimuli in one sensory modality increases activity in cortical regions that process stimuli in the attended modality [18-27]. Additionally, a number of studies also show that selective attention to stimuli in one sensory modality can modulate the unattended sensory cortex by suppressing activity)[19-22,26-28]. For example, Johnson and Zatorre (2006) demonstrated that when participants attended to shapes and ignored melodies, activity increased in visual association cortex and decreased in auditory processing regions compared to conditions where participants passively viewed these same bimodal stimuli. Although these imaging studies suggest an important role for modality-specific attention in modulating activity in both the attended and unattended sensory cortices, it is unknown to what extent modulatory effects are dependent on the presence of a stimulus. That is, decreases in auditory cortex activity during attention to visual stimuli could be produced by the stimulus itself, by top-down modulation of the sensory cortices, or by interactions between bottom-up stimulus properties and top-down attentional processes. In a study by Baier and colleagues (2006), attentional modulation of early auditory and visual cortices was found to be stronger when bimodal stimuli were expected to be associated than when the auditory and visual components of the stimulus were independent. The results of this experiment indicate that modality-specific attention can alter cortical activity prior to the presentation of a target; however the presentation of bimodal stimuli in all trials may have precluded the observation of deactivations in sensory-specific cortices.

During behavioral experiments, prominent performance decrements are observed for targets in the unattended modality. This suggests that an important neural consequence of modality-specific selective attention is the suppression of activity in sensory cortices that process stimuli in the unattended modality. Behavioral experiments that are designed to test performance enhancements and decrements associated with modality-specific attention require the presentation of stimuli in both the attended and unattended sensory modality. However, presenting bimodal stimuli may confound investigations into the physiological effects of modality-specific attention because a bimodal stimulus increases activity in both the attended and unattended sensory modality cortices, thus masking true cross-modal deactivations. To characterize the nature of the neural response to auditory and visual selective attention, this experiment utilized attention cues presented prior to unimodal sensory stimuli. On selected trials, the unimodal target was not presented after the cue, allowing us to explore increases and decreases in primary auditory and visual cortex activity due solely to attention. We hypothesized that suppression of sensory cortices during modality-specific selective attention would be evident in deactivations of the unattended sensory cortex during presentation of the cue alone.

## Methods

### Participants

Fifteen adult volunteers (6 men, 9 women), age 21 to 30 (mean age, 23) participated in this study. All participants were in good health, with normal or corrected to normal vision and normal hearing. After receiving an explanation of the study procedures, all participants provided written informed consent. This study was approved by and conducted in accordance with the Wake Forest University School of Medicine Institutional Review Board for the protection of human subjects.

### Stimuli

Participants completed a cued detection paradigm that included visual and auditory targets designed to asses the effects of modality-specific attention in the presence and absence of target stimuli. In order to ensure that participants would effectively deploy attention on all trials, participants were told that all trials would contain a target following an attentional cue. They were instructed that the purpose of this experiment was to evaluate brain responses to a discrimination task of graded difficulty, so the experiment would include a range of easy to very difficult detection trials. Some auditory and visual targets provided a very small change in stimulus intensity from resting or background levels of stimulation and were very difficult to detect, while others conveyed a larger intensity change and were very salient and easy to detect. Additionally, on 1/3 of the trials, no target was presented after the cue. These "no-target" trials were included to assess the effects of attention, independent of stimulus presentation; however, participants were unaware of their existence as it was difficult to tell no-target trials apart from trials with very faint stimuli. Although including a smaller proportion of no-target trials than target trials reduces the power to detect the effects of the no-target stimulus condition, it was necessary to limit these trials so that participants remained unaware of the absence of a stimulus.

Subjects were instructed to fixate on a white cross in the center of a black screen throughout the experiment. Each trial began with a visually presented cue that provided information about the upcoming target type. A picture of two ears cued subjects to listen for an auditory target. A picture of two eyes cued subjects to look for a visual target. A picture of one eye and one ear cued subjects to pay attention to both vision and audition, as the target could appear in either modality. Additionally, there was a rest cue (two X's) that informed participants to do nothing until the next trial, and a motor cue (two fingers) that prompted subjects to press either button as soon as any target was detected. The no-target trials were different than rest trials, where participants received a rest cue, which alerted subjects that there would be a rest interval instead of a target. The rest and motor and trials were not utilized for any of the analyses included in this paper.

Following the presentation of the cue (500 ms duration), there was a variable delay (1000–1500 ms) before the target stimulus. Each auditory target was a single 50 ms, 500 Hz tone that could vary in volume from 1% to 66% of the volume of a tone adjusted for each participant to a level that could be heard clearly above the scanner noise. Six different volume gradations were created (1%, 5%, 25%, 35%, 50%, and 66% of a suprathreshold stimulus) using the volume reduction function in Goldwave audio editing software . A visual target was a single 50 ms change in the brightness of the black background that could vary in brightness from 5% to 70% (black = 0% brightness, white = 100%). Eight different brightness gradations were created (5%, 10%, 20%, 30%, 40%, 50%, 60%, and 70% of a pure white background) using the lightness function in Photoshop . These graded stimuli were designed to generate a task where participants would have to make present/absent judgments of various difficulty, not to measure precise signal detection properties of each stimulus intensity. The nature of the paradigm, using cues and varying levels of target intensities rather than streams of stimuli at regular intervals, ensures that no-target trials will not elicit an omitted stimulus response.

**Figure 1:**
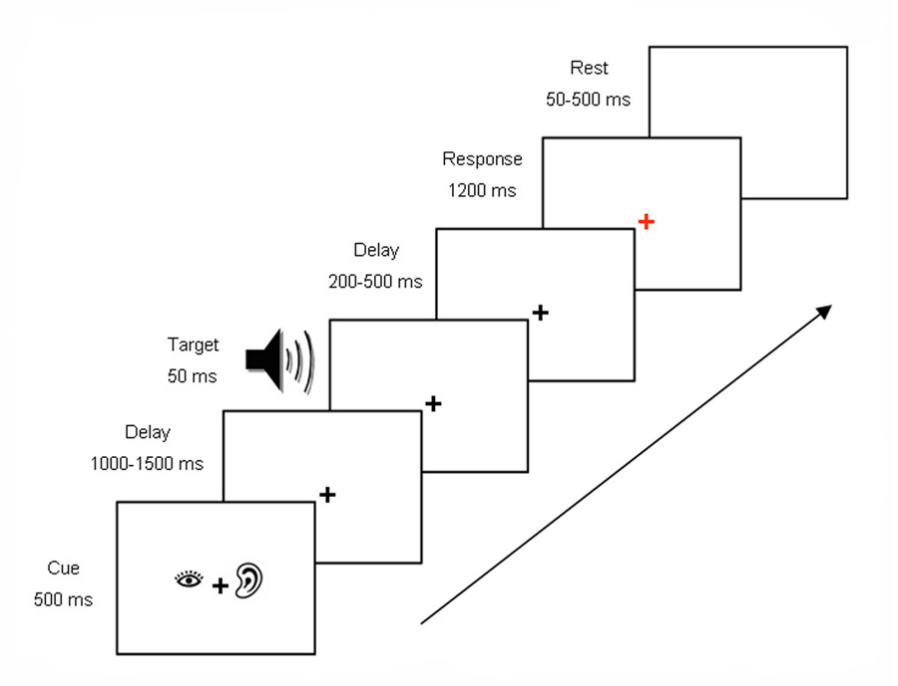
Time course of events in a trial with a divided attention cue and an auditory target.

After the target interval and a variable delay (200 to 500 ms), the white fixation cross in the center of the screen turned red to prompt a response. Participants were instructed to respond by pressing the left button if they perceived a target or the right button if they did not perceive a target. See Figure 1 for a diagram of the time course of trial events. Stimuli were presented using MR compatible goggles and headphones (Resonance Technology, Inc.) with stimulus delivery and response collection controlled by Eprime software (Psychology Software Tools). Participants completed five runs of the task, and each run contained 96 trials presented in random order, for a total of 480 experimental trials. Eighty of these trials were auditory attention/auditory targets, 40 were auditory attention/no target, 80 were visual attention/visual target, 40 were visual attention/no target, 40 were divide attention/auditory target, 40 were divide attention/visual target, and 40 were divide attention/no target. The remainder of the trials were rest or motor trials that were not included in the analyses for this report.

### Image Acquisition

Experiments were performed on a 1.5T GE echo-speed Horizon LX scanner with a birdcage headcoil (GE Medical Systems). High-resolution T1-weighted structural scans were obtained using an inversion recovery 3D spoiled gradient echo sequence (matrix size: 256 × 256, field of view: 24 cm, slice thickness: 3 mm, 128 slices). Whole-brain activation was assessed by examining BOLD signal [29] alterations produced by the changes in blood oxygenation that accompany cortical activation. Functional images were acquired using multi-slice gradient-echo planar imaging (EPI; TR: 2100 ms, TE: 40 ms, matrix size: 64 × 64, slice thickness: 5 mm, 28 slices).

### Analyses

A measure of signal detectiblity, d', was calculated for each attention condition using the hit rate (% of trials where a subject correctly identified that a target was present) and the false alarm rate (% of trials where there was no target but the subject incorrectly responded that one *was *present), in the formula d' = Z_false alarm _- Z_hit_[30]. Although d' analyses are typically performed to evaluate the detectibility of a stimulus of a single intensity, we used the analysis to compare our participants' sensitivity to a range of stimulus intensities across the different attention conditions, while controlling for participants' response biases. Thus our analyses should not be interpreted as a standard signal-detection experiment, but rather as a measure of participants' ability to detect any gradation of the auditory or visual stimuli during selective and divided attention.

The functional images from each subject were reconstructed and processed with SPM99 . Data sets were corrected for slice timing to accurately align images with the time course of the paradigm. To correct for motion, data sets were realigned using the first image as the reference. Images were then normalized to Montreal Neurological Institute space based on the EPI template in SPM99. Images were smoothed using a Gaussian kernel (full width at half maximum, 8 × 8 × 10 mm).

Statistical parametric maps were generated in SPM99 using the general linear model. A regression analysis was performed at each voxel to determine the parameter estimates for each trial type. Each trial in the paradigm was modeled as a single event and the time course of events was convolved with a canonical hemodynamic response function and the time derivative was included. Randomly presented rest trials provided a measure of baseline activity.

Data from the five task runs were combined for each subject in a fixed effects analysis. Contrast weights were applied to the parameter estimates for each trial type, in order to identify regions where the BOLD signal change was correlated with the stimulation paradigm. For example, to identify regions of increased activity during auditory attention trials, a +1 contrast weight was applied to the parameter estimate for that trial type and a 0 contrast weight was applied to all other trial types. Importantly, a -1 contrast weight applied to the parameter estimate for this trial type would identify regions of decreased activity during auditory attention trials. A contrast image representing the sum of the weighted parameter estimates was then generated for each effect of interest. The contrast images for each subject were then used to perform random effects group analyses, where activity between trial types could be compared (e.g., where was activity greater during auditory attention than during visual attention).

Data were globally normalized to allow for group comparisons using proportional scaling of image means and all statistical maps were thresholded at p < 0.001 and corrected for multiple comparisons using extent correction (p < 0.05) unless otherwise noted. In regions identified to have significantly greater activity during one attention condition than another, an ROI (10 mm sphere, centered on the peak voxel in the largest cluster of activation) was drawn to explore the effects driving the differences in activity between the attention conditions. The mean percent signal change in each ROI was then averaged across subjects and compared during each stimulus condition. One sample t-tests were used to determine if signal changes in these ROIs were significantly different from baseline during each stimulus condition, and 2 × 2 repeated measures ANOVAs were conducted to examine the impact of target (present or absent) and attended modality (audition or vision) on signal change in the auditory cortex and the visual cortex.

## Results

### Behavioral Responses

Stimulus detection (d') during the different attention conditions, as well as the hit and false alarm rates used to calculate d', are summarized in Table 1. Participants had increased sensitivity to auditory targets when their attention was directed to the auditory modality (d' = 2.94) versus when their attention was divided between modalities (d' = 2.57), indicated by a significant increase in target detectibility during selective attention trials (t_14 _= 3.52, p < 0.003). The detection analysis did not indicate a significant effect of visual attention on participants' sensitivity to visual targets.

**Table 1:** Average hit rate, false alarm rate, and detectibility (d') with standard deviations (SD) for each cue/target condition.

	Modality-Specific Attention	Divided Attention
Auditory Target		
% hits	81 (2)	74 (4)
% false alarms	3 (1)	4 (1)
d'	2.94 (0.11)	2.57 (0.09)
Visual Target		
% hits	90 (1)	89 (1)
% false alarms	6 (2)	4 (1)
d'	3.08 (0.18)	3.15 (0.13)

### fMRI

**Figure 2:**
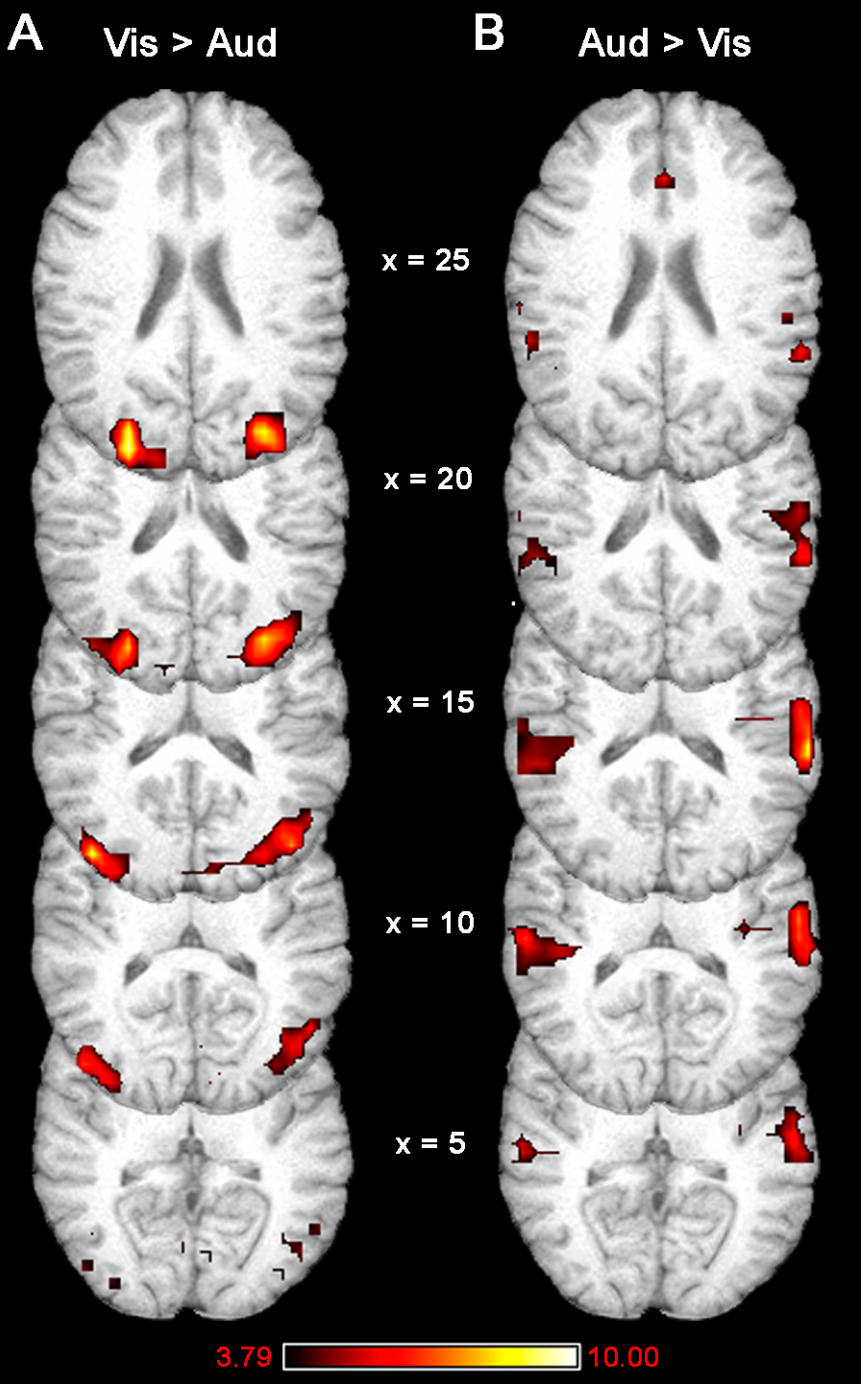
**Greater levels of activity were noted in visual cortex during attend vision trials (Vis) than attend audition trials (Aud; A).** Greater levels of activity were also present in auditory cortex during attend audition trials than attend vision trials (B) Statistical maps are thresholded at p < 0.001 and corrected for multiple comparisons using extent correction (p < 0.05).

During trials where a target was presented after the attention cue, significantly greater levels of activity were noted in the visual cortex during attend vision trials than during attend audition trials (Fig. 2A). Conversely, activity in auditory cortex was significantly higher during attend audition trials than during attend vision trials (Fig. 2B).

**Figure 3:**
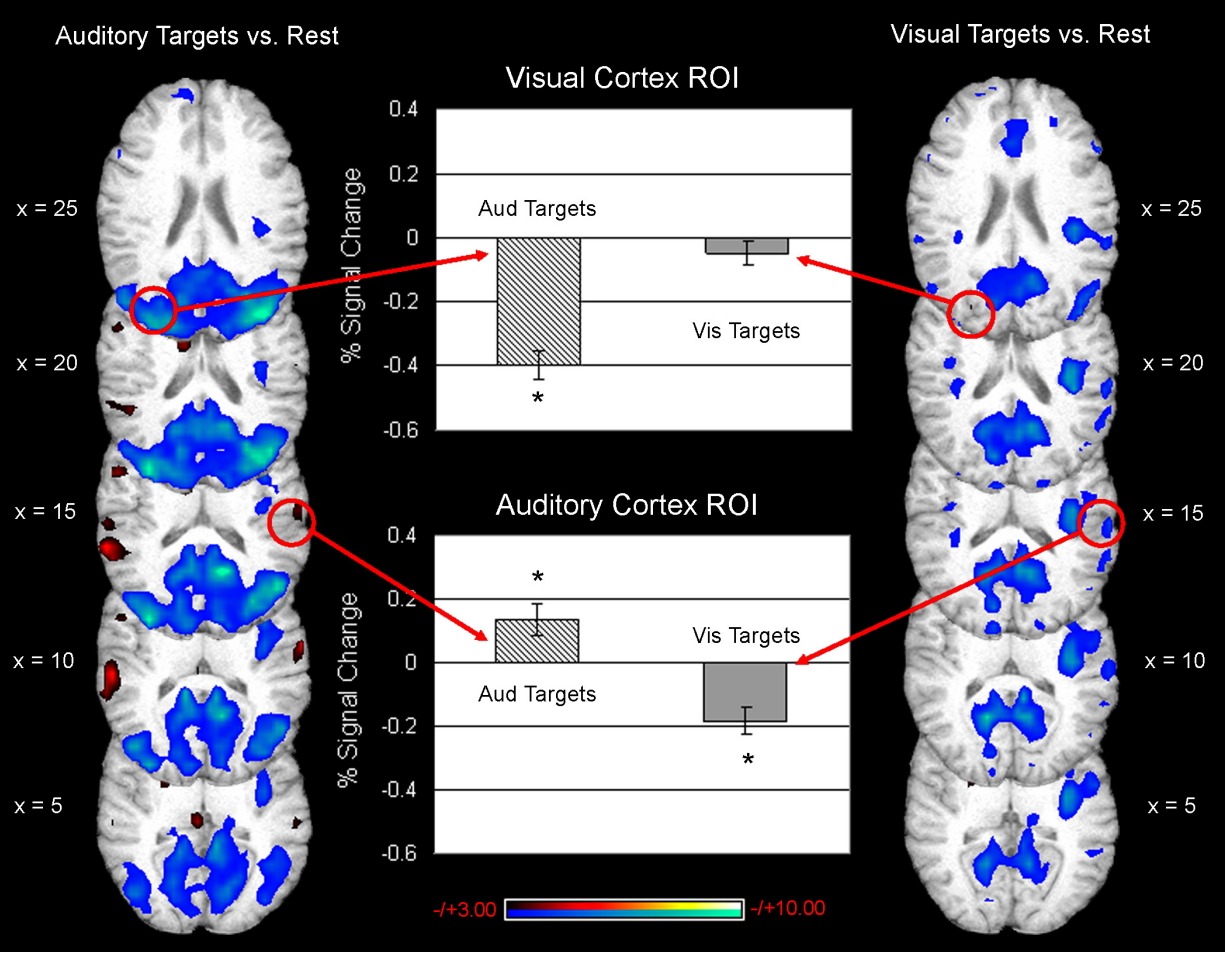
**Examination of sub-threshold (t>3.00, uncorrected) activity during attention to audition (depicted in the left column) or vision (depicted in the right column) indicated that decreases in activity levels in the unattended sensory cortex were the main source of differences between auditory attention and visual attention trials.** Circles indicate the approximate locations of the ROI for peak activity differences between attend audition and attend vision trials (based on results shown in Fig. 2). The % signal change in visual cortex ROI is negative during trials where participants are cued to pay attention to audition and subsequently receive an auditory target (top graph, hatched bar). Very little signal change was noted in this same region of visual cortex when participants were cued to pay attention to vision and subsequently received a visual target (top graph, gray bar). In the auditory cortex ROI, small increases in signal were noted on trials where participants received an auditory attention cue and auditory target (bottom graph, hatched bar). Decreases in signal were also noted in this same region on trials where participants received a visual attention cue followed by a visual target (bottom graph, gray bar). Asterisk (*) indicates a significant change from baseline activity levels, p < 0.05.

These results intuitively make sense; greater visual activity would be expected when a visual stimulus is present than when an auditory stimulus is present. However, further examination using ROI analyses indicated that most of the differences in activity between attend vision and attend audition conditions resulted from activity *decreases *in the unattended sensory cortex. That is, relative increases in visual cortical activity during visual attention were due almost exclusively to *suppression *of visual cortex during auditory attention. It should be noted that the regional analyses for this study were not conducted in primary sensory cortices, but rather in the sensory association cortices where whole brain analyses were used to locate the peak activity differences between visual and auditory attention conditions. The ROI for the visual cortex was centered in dorsal visual association cortex in the occipital lobe (BA 19; MNI 64, -36, 16), and the auditory cortex ROI was located in the auditory association cortex of the superior temporal gyrus (BA 42; MNI 78, 48, 45). Activity decreased below baseline levels in this visual cortex ROI during attend audition trials (mean signal change = -0.40 %, t_14 _= -8.65, p < 0.001), but did not change significantly during the attend vision trials (mean signal change = -0.05 %, t_14 _= -1.29, ns; Fig. 3). Similarly, the relative increases in auditory cortex activity observed when comparing the attend audition trials to attend vision trials were driven primarily by significant *deactivations *in auditory cortex during attend vision trials (mean signal change = -0.19 %, t_14 _= -4.45, p < 0.001; Fig. 3). Small activations noted in auditory cortex during attend audition trials also contributed to the relative differences observed between attend audition and attend vision conditions (mean signal change = 0.14 %, t_14 _= 2.82, p < 0.014; Fig. 3).

The subtle stimuli used as targets in this experiment did not produce large positive changes in the BOLD signal, so it is unlikely that they were the only factors driving the deactivations in unattended sensory cortices. However, the presence of the target in these trials does not allow the relative contributions of sensory- and attention-driven deactivations to be ascertained. Therefore, sensory responses to "no-target" trials were also analyzed.

**Figure 4:**
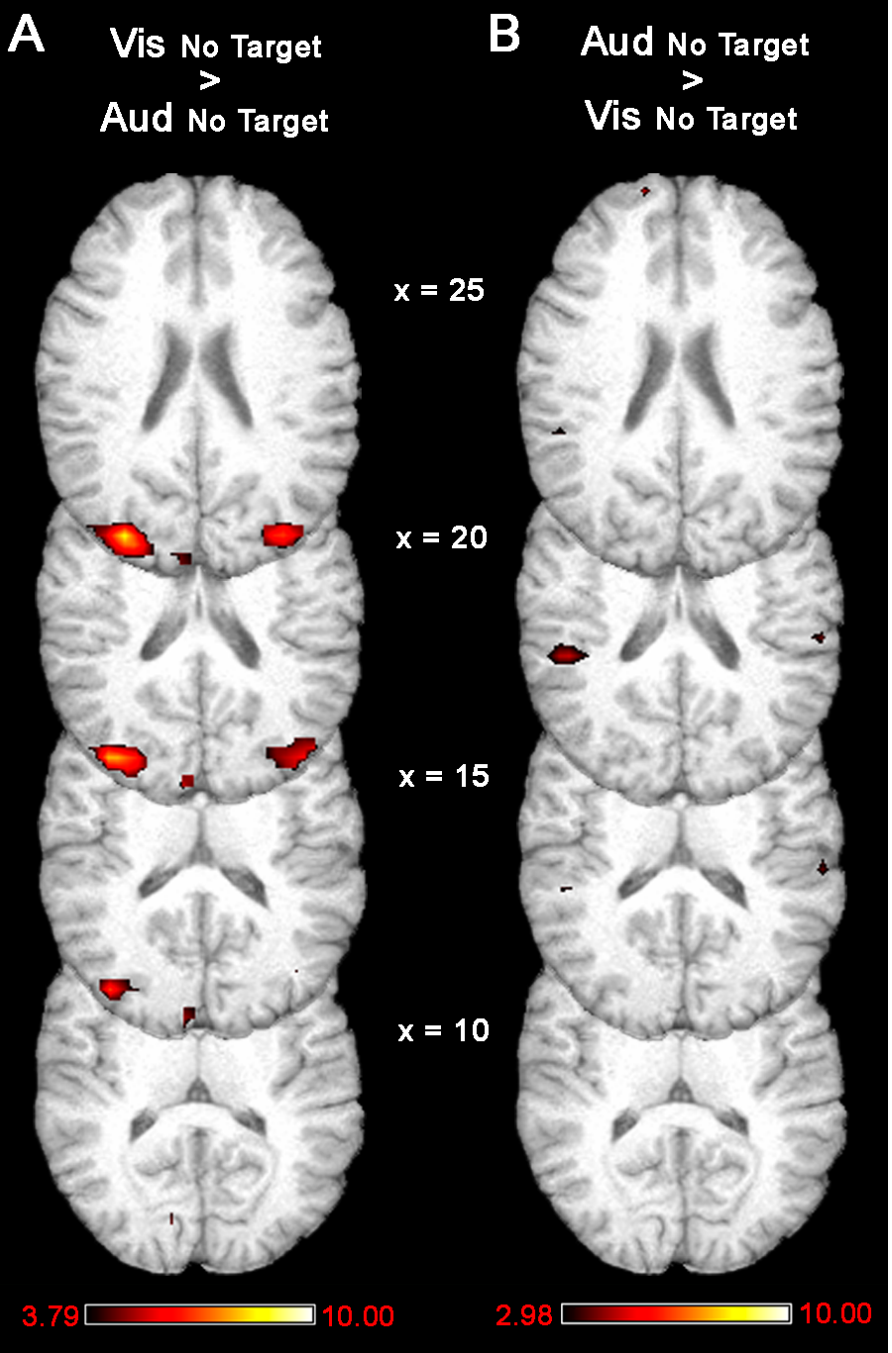
**Comparisons between trials where no target was presented after the attention cue demonstrate that there were greater levels of activity in visual cortex during attend vision trials than attend audition trials (using a correction threshold of p < 0.001, cluster extent p < 0.05; A).** Greater levels of activity were also present in auditory cortex during attend audition trials than attend vision trials (using a correction threshold of p < 0.005, cluster extent p < 0.05; B).

Activity was compared between the attend audition trials and the attend vision trials when no stimulus was presented after the cue in order to verify that attention alone, in the absence of any stimulus, could cause cross-modal deactivations (Fig. 4). Due to the limited number of no-target trials, the power of this comparison was considerably reduced. Nevertheless, significantly more activity was observed in visual cortex when visual attention was compared to auditory attention in the absence of an actual sensory stimulus (Fig. 4A). The comparison of differences in the auditory cortex during auditory and visual attention for no-target trials did not survive the correction for multiple comparisons in a whole brain analysis, but whole brain maps are presented for illustrative purposes (Fig. 4B). Region-specific analyses were also performed to explore the activity differences during visual and auditory attention.

**Figure 5:**
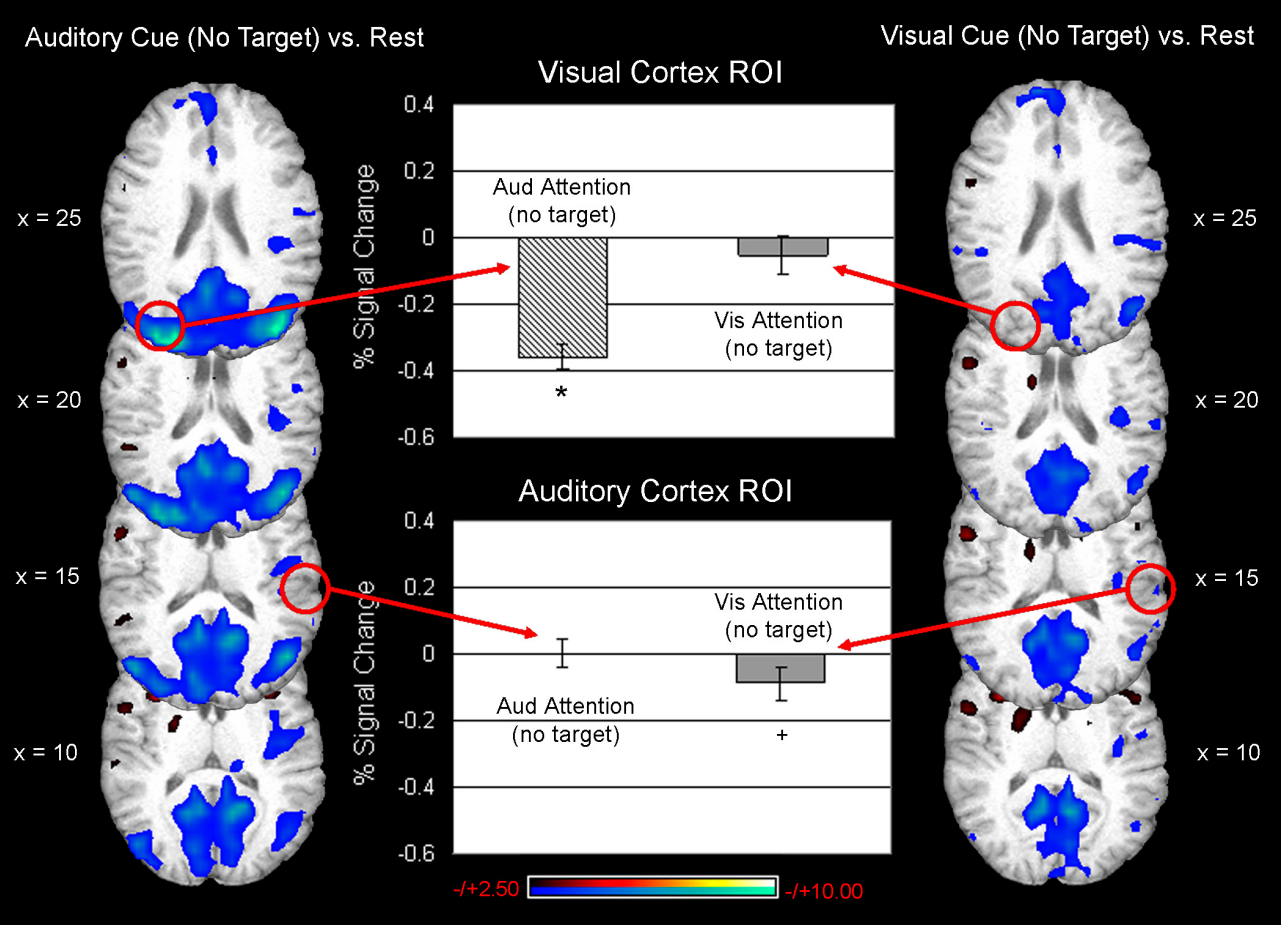
**Examination of sub-threshold (t> 2.50, uncorrected) activity during attention to audition (depicted in the left column) or vision (depicted in the right column) indicated that decreases in activity levels in the unattended sensory cortex were the main source of differences between the auditory attention trials and the visual attention trials where no target followed the attention cue.** Circles indicate the approximate locations of the ROI for peak activity differences between attend audition and attend vision trials (based on results shown in Fig.2). The % signal in visual cortex ROI decreased significantly below baseline during trials where participants are cued to pay attention to audition but received no auditory target (top graph, hatched bar). No significant signal change was noted in this same region of visual cortex when participants were cued to pay attention to vision but subsequently received no visual target (top graph, gray bar). In the auditory cortex ROI, no change in signal was noted on trials where participants received an auditory attention cue but no auditory target (bottom graph, hatched bar). However, slight decreases in signal were noted in this same region on trials where participants received a visual attention cue but no visual target (bottom graph, gray bar). Asterisk (*) indicates a significant change from baseline activity levels (p < 0.05); cross (+) indicates a trend towards a change from baseline activity (p < 0.10).

As observed in conditions where a stimulus followed the cue, the "increases" in the attended cortex during no-stimulus trials were actually due to activity *decreasing *below baseline levels in the unattended sensory cortex. In fact, no significant increases were observed in visual cortex during visual attention (mean signal change = -0.05 %, t_14 _= -0.89, ns) or auditory cortex during auditory attention (mean signal change = 0.004 %, t_14 _= 0.09, ns) when no stimulus was presented (Fig. 5). Increases observed in visual cortex during attend vision relative to attend audition trials were due to activity decreases below baseline levels in the visual cortex when subjects were cued to attend to audition, despite the fact that no auditory target was presented (mean signal change = -0.36 %, t_14 _= 9.72, p < 0.001; Fig. 5). Additionally, sub-threshold increases in auditory cortex noted when comparing attend audition to attend vision trials were actually due to slight suppression of auditory cortical activity during attend vision trials (mean signal change = -0.09 %, t_14 _= -1.68, p < 0.10), not increases in auditory activity during attend audition trials (Fig. 5).

**Figure 6:**
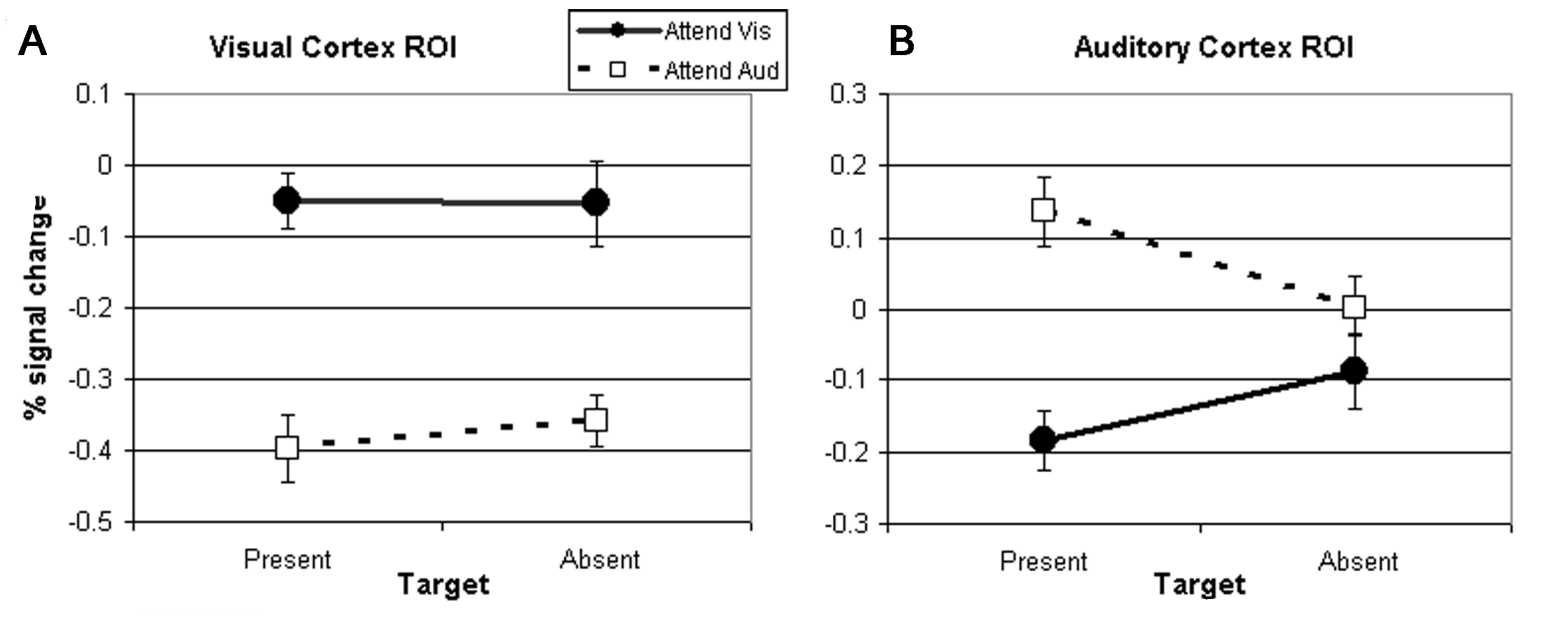
**Analysis of activity in the visual cortex indicated that there was significantly greater deactivation during attention to the auditory modality than during attention to the visual modality, and that the absence of a target did not diminish this effect (A).** In the auditory cortex, there was significantly less activity during visual attention than during auditory attention (B). This effect was modulated by the target such that the absence of a target reduced the magnitude of both the activation observed during auditory attention and the deactivation observed during visual attention (B).

To further explore the impact of target and attention on signal change in the sensory cortices, we conducted separate 2 _target status _× 2 _attended modality _repeated measures ANOVAs on activity in the visual cortex ROI and the auditory cortex ROI (Fig. 6). For the visual cortex, the attended modality had a significant impact on activity (F_1,14 _= 95.35, p < 0.001), but there was no effect of target status (F_1,14 _= 0.233, ns), and no interaction (F_1,14 _= 0.913, ns). These results indicate that there was a larger decrease in activity in the visual cortex during auditory attention than during visual attention, and that this deactivation was not diminished in the absence of a target (Fig. 6A).

In the auditory cortex, there was also a significant effect of attended modality (F_1,14 _= 24.80, p < 0.001), and no effect of target status (F_1,14 _= 0.26, ns), but there was a significant interaction of attention and target (F_1,14 _= 19.29, p < 0.001). This analysis demonstrates that there was less activity in the auditory cortex during visual attention than auditory attention, and that this activity (both activation during auditory attention and deactivation during visual attention) was reduced in magnitude in the absence of a target (Fig. 6B).

## Discussion

Attention is a neural mechanism for selecting behaviorally relevant information from the multitude of stimuli encountered in the environment. Data from psychophysical investigations suggest that the primary behavioral gains of modality-specific attention come from suppression of responses to stimuli in the unattended modality rather than enhancing responses to attended stimuli. Results of the present fMRI study seem to reflect the neural underpinnings of this phenomenon, as the primary difference between attending to vision and audition was decreased neural activity in the unattended sensory modality rather than enhancement of activity in the attended modality. Notably, this attentional modulation was also observed on trials when no target was presented following the attentional cue. Studies exploring visual spatial attention have demonstrated a similar effect, whereby simply directing attention to a particular location modulates sensory cortex activity, independent of stimulus presentation [14,31].

Suppression of visual cortex during auditory attention was not diminished in the absence of a target; however, the magnitude of activity increases and decreases in the auditory cortex was reduced when no target was presented in the attended modality. Sub-threshold deactivations of auditory cortex were still noted during visual attention no-target trials, indicating that there may be an additive effect between top-down components of attention and bottom-up influences of sensory stimulation that occurs when a cue is followed by a target. Smaller deactivations in auditory cortex during the no- target conditions could reflect the absence of this additive effect. The visual stimulus used in this paradigm did not produce significant activation in the visual cortex; however, future experiments using more effective visual stimuli might allow the observation of increased stimulus-driven deactivations in the auditory cortex as well as activations in the visual cortex. Additionally, utilizing different visual stimuli would likely activate visual cortex regions not encompassed by the ROI analyzed in this experiment. Other regions of visual cortex may show different relationships between stimulus and attention-driven modulations.

An alternative explanation for reduced deactivations of auditory cortex during no-target conditions is that our experimental design required there to be fewer no-target trials than trials where the target is present, which may have reduced the detectibility of attention-mediated changes in activity that are independent of stimulus presentation. Future studies containing a greater proportion of no-target trials may shed further light onto this finding.

Our imaging results are in accord with data from behavioral studies demonstrating that modality-specific attention generally produces large behavioral decrements for tasks in the unattended sensory modality, but only small benefits for tasks in the attended modality [16,21]. While our experimental design precluded the observation of behavioral decrements, we were able to measure small, but significant behavioral improvements associated with auditory attention. Future studies utilizing alternative experimental designs will be required to explore the neural events associated with behavioral performance decrements.

Importantly, however, the behavioral enhancements resulting from selective auditory attention were reflected in the neural activity changes observed in both auditory and visual cortices during auditory attention. Behavioral benefits of visual attention were only apparent in speeded response times, not enhanced detection, and the neural activity changes corresponding to visual attention are not as robust as those for auditory attention. One possible source of differences between results for the two modalities is that the visual and auditory environments in the MR scanner are somewhat disparate due to the acoustic noise generated by rapid gradient switching. Thus, when auditory stimuli were presented in the midst of ongoing scanner noise, increased selective attention may have been required. In contrast, when the visual stimuli were presented, there was little competing visual noise, possibly requiring less engagement of selective attention mechanisms. Another potential explanation for the divergence in auditory and visual results is that deactivations in the visual cortex observed in the absence of an auditory target could due to the continuous auditory noise in the scanner environment. That is, scanner noise could be acting as an ongoing auditory signal, producing deactivations in visual cortex indistinguishable from those produced by the auditory target tone used in this paradigm. To equate the visual and auditory environments more closely, future studies could use sparse or clustered temporal acquisition fMRI techniques to present auditory stimuli without the presence of background scanner noise [32].

In contrast to previous reports [18-21,27], attention to a particular modality during our task did not increase activity in the attended sensory cortex. In each of these previous studies, however, a salient stimulus was always present in the attended modality. The stimuli used in our detection paradigm were, by necessity, often nearly imperceptible and, at times, completely absent. Thus the increases observed in the attended modality for previous studies were likely due to enhanced responses to attended stimuli, not increases in baseline activity in the attended modality.

Interactions between stimulus salience and attentional modulation of both attended and unattended sensory cortices are consistent with data from Shulman *et al*. (1997). They demonstrated that in paradigms producing a weak response to passive sensory stimulation, there was also little modulation during active attention to the same sensory stimuli. In contrast, robust attentional modulation of attended stimuli was observed for stimulus conditions that produced a strong sensory response during passive viewing [27]. Rinne and colleagues (2005) also demonstrated an interaction between auditory stimulus presentation rate and attentional modulation. Increasing sound presentation rate and focusing attention on the auditory modality both increased auditory cortex activity, however, attentional effects are enhanced at higher sound presentation rates [24].

In summary, our data demonstrate that modality-specific selective attention produces activity decreases in the ignored sensory modality and not 'true' increases in cortical activity for the attended modality. These data are consistent with behavioral experiments demonstrating that performance enhancements during modality-specific selective attention result primarily from decreased processing in the unattended modality not increased processing in the attended modality. Furthermore, this experiment demonstrates that deactivations of cross-modal cortices noted during sensory stimulation can be produced exclusively by endogenous focusing of attention on the target modality and are not solely dependent on the presentation of a stimulus. However, the data suggest an additive effect of attentional and stimulus-driven effects. Activity increases in the attended modality have been previously reported when subjects are actively processing highly salient stimuli. In our work we did not see a significant increase in baseline activity in the attended modality, suggesting that activations in the attended modality are likely dependent on an interaction between attention and the presence of a salient stimulus. Such an interpretation of the effect of modality-specific selective attention is reasonable given that there would be no behavioral benefit of increasing background activity in the absence of a stimulus, but there would be a benefit of enhancing a response to a particular stimulus.

## Competing interests

The authors declare that they have no competing interests.

## Authors' contributions

JLM participated in data analysis and wrote the manuscript. DJ participated in designing the experiment, data collection, and data analysis. CEH participated in designing the experiment and data collection. AMP participated in designing the experiment and data collection. RAK developed the MRI sequences and protocol. JAM developed the data processing software. PJL participated in designing the experiment, data analysis, and manuscript revision.

## Pre-publication history

The pre-publication history for this paper can be accessed here:


